# Backbone chemical shift spectral assignments of SARS coronavirus-2 non-structural protein nsp9

**DOI:** 10.1007/s12104-021-10011-0

**Published:** 2021-03-23

**Authors:** Erika F. Dudás, Rita Puglisi, Sophie Marianne Korn, Caterina Alfano, Maria Laura Bellone, Fabrizio Dal Piaz, Geoff Kelly, Elisa Monaca, Andreas Schlundt, Harald Schwalbe, Annalisa Pastore

**Affiliations:** 1 Department of Basic and Clinical Neuroscience, Maurice Wohl Institute, UK-DRI at King’s College London, 5 Cutcombe Rd, London, SE59RT UK; 2 Institute for Molecular Biosciences, Johann Wolfgang Goethe-University Frankfurt, Max-von-Laue-Str. 9, 60438 Frankfurt/M, Germany; 3 Institute for Organic Chemistry and Chemical Biology, Center for Biomolecular Magnetic Resonance (BMRZ), Johann Wolfgang Goethe-University Frankfurt, Max-von-Laue-Str. 7, 60438 Frankfurt/M, Germany; 4 Fondazione Ri.Med, 90133 Palermo, Italy; 5 Department of Medicine and Surgery, University of Salerno, Via Giovanni Paolo II, 84081 Baronissi, SA Italy; 6 Francis Crick Institute, MRC Biomedical NMR Centre, 1 Midland Rd, London, NW1 1AT UK

**Keywords:** Coronavirus, Covid-19 NMR, Protein, SARS-CoV-2, Solution NMR, Structure

## Abstract

As part of an International consortium aiming at the characterization by NMR of the proteins of the SARS-CoV-2 virus, we have obtained the virtually complete assignment of the backbone atoms of the non-structural protein nsp9. This small (12 kDa) protein is encoded by ORF1a, binds to RNA and seems to be essential for viral RNA synthesis. The crystal structures of the SARS-CoV-2 protein and other homologues suggest that the protein is dimeric as also confirmed by analytical ultracentrifugation and dynamic light scattering. Our data constitute the prerequisite for further NMR-based characterization, and provide the starting point for the identification of small molecule lead compounds that could interfere with RNA binding and prevent viral replication.

## Biological context

The severe acute respiratory syndrome coronavirus 2 (SARS-CoV-2) infection is already the third coronavirus infection that has occurred since the start of the third millennium. This third epidemic has plagued the world population over almost all of 2020 and is still actively on-going. The current situation has thus underlined the importance of gaining a deep and lasting understanding of the rules that regulate viral internalization and reproduction and the necessity to translate this knowledge into a vaccine and/or other treatments. SARS-CoV-2 is an RNA virus comprising a large single-stranded positive polarity genome that acts as messenger RNA after entering the host (Wu et al. 2020; Masters and Perlman 2013). The 5′ two-third of the genome encodes a long polyprotein that is translated in two open reading frames, ORF1a and ORF1b, through host ribosomal frameshifting. The viral RNA also encodes a few structural and accessory proteins within smaller ORFs. As in all viruses, the SARS-CoV-2 genome contains all the proteins necessary for host cell infection such as the RNA polymerase along with enzymes that facilitate RNA synthesis. These proteins are released by the action of two internally encoded proteases. The mature proteins are referred to as non-structural proteins (nsps) as they do not per se constitute the virion shield.

As a part of an International consortium aimed at the study of all SARS-CoV-2 proteins by nuclear magnetic resonance (NMR), this paper describes the nearly complete backbone NMR resonance assignment of the non-structural protein 9 (nsp9). This 12 kDa protein, which is encoded in ORF1a, is a replicase that has been shown to be essential for replication (Frieman et al. 2012; Littler et al. 2020). This feature makes nsp9 a potential target for drug discovery aimed at inhibiting viral replication. Nsp9 forms discrete foci in the perinuclear region of infected cells and colocalizes with other components of the viral replication complex (Frieman et al. 2012). The structures of several homologues of nsp9 are available from SARS-CoV (Yang et al. 2003), the transmissible gastroenteritis (Anand et al. 2002), human corona 229E (Anand et al. 2003), avian infectious bronchitis, porcine epidemic diarrhea, and porcine delta viruses. The crystal structure of nsp9 from SARS-CoV-2 was also recently published (Littler et al. 2020). Availability of the crystal structure does not, nevertheless, reduce the interest of studying the protein in solution as this is the prerequisite to fragment based drug screening and other experimentally-based drug design strategies. Nsp9 proteins have a fold that vaguely resembles that of the oligonucleotide/oligosaccharide binding (OB) domain (Sutton et al. 2004). Nsp9 is an RNA-binding protein that interacts with and activates other proteins of the viral cascade (Sutton et al. 2004). The mechanism of RNA binding within the nsp9 protein family is not understood as these proteins have an unusual structural fold not previously seen in RNA-binding proteins (Egloff et al. 2004; Sutton et al. 2004). In all crystal structures, the protein consistently forms a dimer with an interface mediated by a conserved ‘‘GxxxG’’ motif in the C-terminal-helix. A dimeric form is thought to be critical for viral replication (Miknis et al. 2009). Disruption of key residues in the ‘GxxxG’ motif reduces both RNA binding (Sutton et al. 2004) and SARS-CoV viral replication (Frieman et al. 2012).

Our data provide the prerequisite for further studies of nsp9 by NMR and will help in the identification of small molecules able to interfere with RNA and other partner binding and, thus, to stop viral replication.

## Methods and experiments

### Construct design

The amino acid sequence of SARS-CoV-2 nsp9 was obtained from NCBI reference entry YP_009725305.1 (Wu et al. 2020). Domain boundaries were defined according to the available crystal structures of SARS-CoV-2 nsp9 (PDB codes 6w4b and 6w9q). The sequence encoding for amino acids 1 to 113 (corresponding to full length nsp9) was codon-optimized for *E. coli* expression. The gene was obtained from GenScript Biotech (Netherlands), inserted into the pET3b-based vector pKM263, as well as pET21b( +)-based vector pET-TEV-Nco. Vector pKM263 encodes for an N-terminal His_6_-tag and a GST-tag, followed by a TEV cleavage site, while pET-TEV-Nco only included an N-terminal His_7_-tag and the TEV cleavage site. After proteolytic cleavage (same for both vectors), the 12.4 kDa protein contained four artificial residues (Gly, Ala, Met and Gly), before the start of the native protein sequence.

### Protein production

SARS-CoV-2 nsp9 was cloned and recombinantly expressed in *E. coli.* Two independent preparations were carried out by different teams. In the first, uniformly ^13^C,^15^N-labelled nsp9 was obtained from a BL21(DE3) culture (induction with 1 mM IPTG, for 20 h at 22 °C) adding to the medium 0.5 g/l ^15^N ammonium chloride and 2 g/l ^13^C d-glucose for labelling. It was purified by Immobilized Metal Affinity Chromatography (IMAC) on a Ni^2+^-NTA gravity flow column (Sigma-Aldrich) in 50 mM Tris–HCl, at pH 8, 300 mM NaCl, 10 mM Imidazole, 4 mM DTT and eluted between 150 and 500 mM imidazole. The GST-tag was cleaved overnight by TEV protease (0.5 mg of TEV protease per 1 L of culture) dialyzing in the same buffer used for IMAC, followed by a reverse Ni^2+^-NTA in the same buffer, as reported for SARS-CoV nsp9 (Sutton et al. 2004). Further purification was carried out by size exclusion (SEC) performed on a HiLoad 16/600 Sephadex 75 pg column (GE Healthcare) in SEC buffer (25 mM sodium phosphate, 150 mM NaCl, 2 mM TCEP, 0.02% NaN_3_, pH 7). Nsp9 eluted mainly as a dimer, as estimated by its elution volume. In addition, higher oligomeric species of nsp9 were observed, eluting earlier from the column, that showed significantly increased A260/280 ratios (> 1) and could not be highly concentrated (< 2 mg/mL). Pure nsp9 containing fractions (of the supposedly dimeric species) were determined by SDS-PAGE and pooled. A portion of this sample, called hereafter sample A, was kept at a low concentration of 180 µM to avoid potential induction of oligomerization. Another portion was concentrated to 410 µM, yielding sample B.

A similar protocol was followed in the second preparation scheme with minor adaptations. Both unlabelled and uniformly ^13^C,^15^N-labelled samples were prepared. The protein was purified by IMAC in 25 mM sodium phosphate at pH 7.4, 150 mM NaCl, 1 mM DTT, and 20 mM imidazole and then eluted from the Nickel column with a 0% to 100% gradient of 25 mM sodium phosphate at pH 7.4, 150 mM NaCl, 1 mM DTT, and 400 mM Imidazole. TEV cleavage was obtained by overnight incubation of TEV at a protein:enzyme ratio of 50:1. The cleaved protein was recovered by reverse IMAC and further purified by SEC in 25 mM sodium phosphate, 150 mM NaCl, 2 mM TCEP, pH 7 using a HiLoad 16/600 Sephadex 75 pg column (GE Healthcare). For the unlabelled sample, SEC runs were carried out at different protein concentrations to check if the SEC profile changed as a function of concentration. No difference was observed. SEC fractions of the uniformly ^13^C,^15^N-labelled nsp9 sample were pulled together and concentrated from 100 µM up to 650 µM (sample C).

### Mass spectrometry-based analysis of amino acid sequence

The identity of nsp9 was verified by a mass spectrometry-based peptide mapping approach. Sample A and an unlabelled version of sample C were loaded on a 12.5% SDS polyacrylamide gel. The resulting band underwent trypsin-catalyzed in-gel digestion. NanoUPLC-hrMS/MS analysis of the sample was carried out on a Q-Exactive orbitrap mass spectrometer (Thermo Fisher Scientific, Waltham, MA, USA), coupled with a nanoUltimate300 UHPLC system (Thermo Fisher Scientific). Peptides separation was performed on a capillary EASY-Spray PepMap column (0.075 mm × 50 mm, 2 µm, Thermo Fisher Scientific) using aqueous 0.1% formic acid and CH_3_CN containing 0.1% formic acid as mobile phases and a linear gradient from 3 to 40% of B in 45 min and a 300 nL·min^−1^ flow rate. Mass spectra were acquired over an m/z range from 375 to 1500. To achieve protein identification, MS and MS/MS data underwent Mascot software (v2.5, Matrix Science, Boston, MA, USA) analysis using the non-redundant UniprotKB/Swiss-Prot database (Release 2020_03).

### NMR experiments

The spectra of the samples A, B and C from the two independent preparations were recorded and analysed. The samples were studied in 25 mM sodium phosphate buffer at pH 7.0, 150 mM NaCl, 2 mM TCEP, 0.02% NaN_3_. For comparison, the assignment of a triply labelled ^13^C, ^15^N and ^2^H protein of a close homologue (97% sequence identity) from SARS-CoV (BMRB entry 6501) had been obtained in 50 mM sodium phosphate at pH 6.8, 50 mM NaCl. By default, 10% D_2_O was added to the final NMR samples. Condition screenings were done in the range of temperatures 278–308 K and of pH 5–7. In the end, the best S/N ratio was observed at 298 K and pH 7.0. At lower pH we observed only a few additional HSQC cross-peaks. NMR spectra were recorded on Bruker spectrometers working at 700, 800 and 950 MHz and equipped with cryo-probes. Water suppression was achieved with the WATERGATE pulse sequence (Piotto et al. 1992). For the sequential backbone resonance assignment, a set of 3D NMR experiments was used: HNCO, HN(CA)CO, HNCA, HN(CO)CA, HNCACB and ^15^N-edited NOESY-HSQC. Non-uniform sampling was employed, with the number of points in indirect dimensions set to ~ (0.66 * 2^N) and extended to 2^N in SMILE (Ying et al. 2017). The schedules were exponentially weighted with a time constant equal to the acquisition time in that dimension. Selected parameters are listed in Table 1.

**Table 1:** Summary of the NMR experiments recorded

	TD			SW(ppm)			MHz	NS	NUS (%)
Sample A									
HSQC	2048	400		14	31		700	16	–
HNCO	2048	84	84	14	31	14	700	8	25
HN(CA)CO	2048	84	84	14	31	14	700	48	29
HNCA	2048	84	84	14	31	30	700	64	25
HN(CO)CA	2048	84	84	14	31	30	700	64	25
HNCACB	1024	60	140	14	31	74	800	16	–
N15-NOESY	2048	84	340	14	31	12	800	8	50
Sample B									
HSQC	3072	400		14	31		950	16	–
HNCO	1536	84	84	14	31	14	700	40	25
HN(CA)CO	1536	84	84	14	31	14	700	104	25
HNCA	1536	84	84	14	31	30	950	64	45
HN(CO)CA	1536	84	84	14	31	30	950	40	45
HNCACB	2048	84	168	14	31	80	950	64	28
N15-NOESY	1536	84	340	14	31	12	950	32	27

All NMR spectra were processed using the NMRPipe/SMILE (Delaglio et al. 1995; Ying et al. 2017) and analyzed using CCPNMR (Skinner et al. 2016)

### Assignments and data deposition

**Fig. 1:**
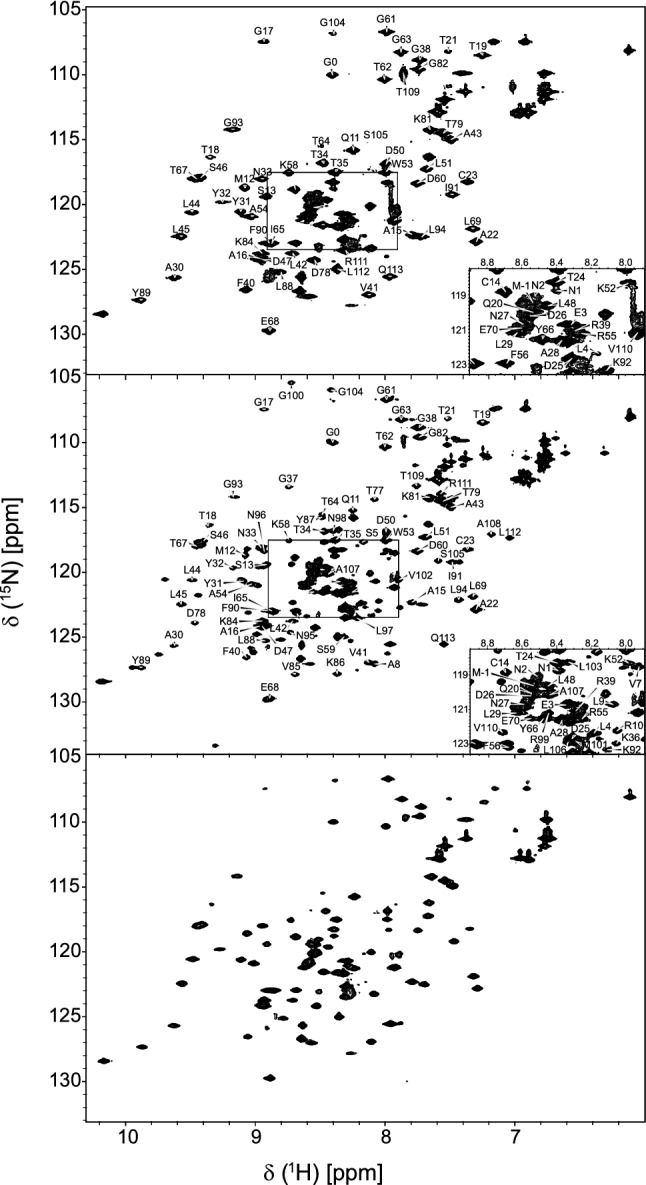
Comparison of spectra recorded at three different protein concentrations. Spectra of samples **a** 180 µM, top, **b** 410 µM, middle and **c** 600 µM, bottom recorded at 700 MHz, 950 MHz and 800 MHz respectively. Spectra of sample A and B were annotated with the assignment. Notice that, for clarity, the counter levels were chosen to exclude noise. Thus, not all of the duplicated peaks observed in the spectrum of sample B are visible

The identity and integrity of the samples were tested by trypsin hydrolyses supported by high-resolution LC/MS–MS analysis. The backbone chemical shift assignment of nsp9 was carried out manually using standard double- and triple-resonance NMR experiments on uniformly labelled samples and recorded at 298 K. Despite the overall good quality of the spectra obtained, assignment of nsp9 presented some challenge. The number of signals observed in the ^1^H-^15^N HSQC experiments for the less concentrated sample A was 112, including sidechain amides. This number was significantly lower than the expected resonances considering 111 non-proline residues, one indole of a tryptophan and twelve side chains of glutamines and asparagines. Conversely, the number of resonances was approximately 50% higher than expected for the more concentrated sample B (Fig. 1). We originally interpreted this difference with the presence of an equilibrium between a monomeric and dimeric species in a slow exchange rate regime. However, this interpretation would disagree with previous literature on nsp9 both from SARS-CoV-2 and other coronaviruses (Littler et al. 2020; Sutton et al. 2004). These studies have all reported that the protein is present in solution as an obligate dimer above 100 µM whose dimer interface involves a parallel packing of the C-terminal helices (Sutton et al. 2004).

Later preparation of sample C clarified the issue: aliquots from the same preparation were increasingly concentrated from 100 to 650 µM and HSQC spectra recorded. The resulting spectrum of sample C was independent of concentration and perfectly superimposable with that of sample A, indicating that this must be considered the spectrum representative of the dimeric form of this protein. Since the spectrum of this sample did not contain any new information it was kept only for comparison and not further analysed. Consequently, sample B must correspond to an alternative second conformation. Both the spectra of samples A and B, recorded at neutral pH, remained stable for at least four months.

^1^H, ^15^N and ^13^Cα chemical shifts of a close homologue (three residues different) from SARS-CoV deposited in the BMRB (entry 6501) were transferred to ^1^H-^15^N HSQC, HNCA, and HN(CO)CA spectra recorded for sample A. Isolated resonances were identified first in the HSQC spectrum using the CCPNMR software, then assignment involving residues in the overlapping region was performed based on Cα shifts. Limited magnetization transfer constrained the use of the HNCACB strips only to resolve specific ambiguities of the HNCA.

Approximately 30 non-proline residues of sample B, most of them corresponding to residues in the C-terminal helix, were not observed in samples A and C despite mass spectrometry analysis had confirmed the presence and the correct molecular weight of all of the nine peptides expected from digestion, representing 99% of the protein sequence. In sample B, only residues Q49, C73, R74, F75, and V76 were not assigned. The C-terminal helix (N95-R111) could be fully assigned in this sample based on the HNCA spectra with the support of characteristic cross-peak patterns observed in strips in the ^15^N-edited NOESY-HSQC.

Consequently, we could assign in the end 73% of the nitrogen atoms, 73% of the amide protons, 77% of the Cα, 41% of the Cβ and 76% of the C’ resonances for sample A. For sample B, 95% of the nitrogen atoms, 95% of the amide protons, 96% of the Cα, 63% of the Cβ and 96% of the C’ resonances were assigned.

The assignments of both sample A and B were deposited in BMRB with accession numbers 50622 and 50621 respectively. They were overall in excellent agreement with each other except for the assignment of the C-terminus where main differences with sample B were evident at the few residues observable in sample A. Our assignment was also in good agreement with that of the SARS-CoV nsp9 homologue, especially for the Cα carbons which are nuclei less affected by the environmental conditions (pH, buffer content and perdeuteration). The larger differences were observed again in the assignment of the C-terminal helix.

### Structural comparison of nsp9 from SARS-CoV2 and its crystal structure

**Fig. 2:**
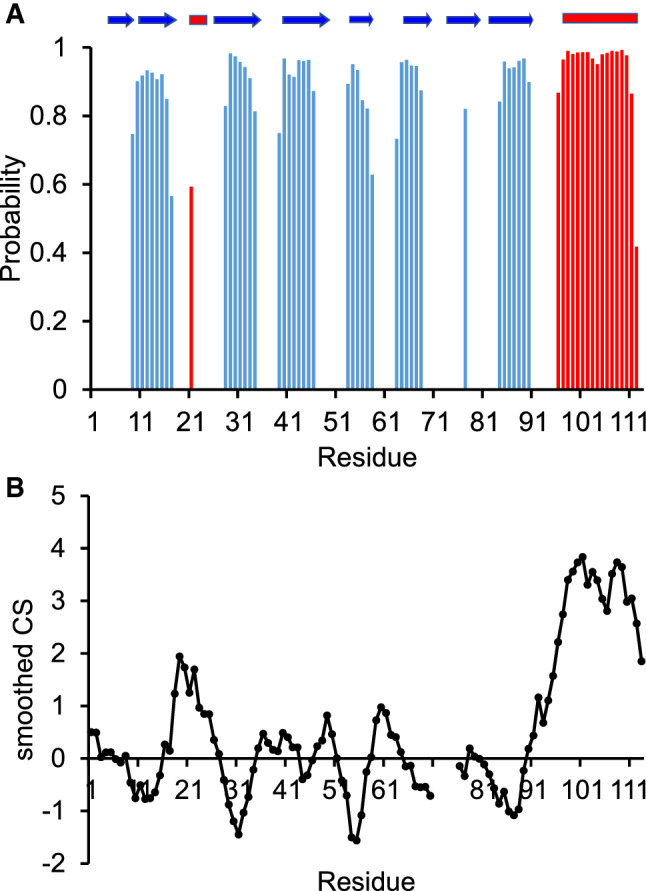
Secondary-structure prediction of nsp9 based on the backbone chemical shifts of sample B. **a** TALOS_N prediction. On the top is, for reference, the schematic indication of the secondary structure elements observed in the X-ray structures (PDB codes 6w4b and 6w9q). **b** Prediction obtained by smoothing the secondary chemical shifts (observed chemical shifts – random coil chemical shifts) smoothed according to Pastore and Saudek (1990). The smoothing window was ± 2

The backbone resonance assignment was used to predict secondary structure elements of nsp9 by TALOS-N (Shen et al. 2013). We obtained a plot which clearly indicated the presence of seven beta strands and two helices (Fig. 2a**)**. We also adopted for comparison the approach by Pastore and Saudek (1990) in which the secondary Cα carbon chemical shifts are smoothed and plotted versus the protein sequence (Fig. 2b**)**. The experimentally determined structural elements were compared with motifs obtained from the X-ray structures of SARS-CoV nsp9 (PDB codes 6w4b and 6w9q). Overall, the structural elements of the proteins are fully consistent reproducing seven beta strands out of the eight expected, a C-terminal helix and a short one-turn 3_10_ helix at residues 22–24.

### Surface of dimerization

**Fig. 3:**
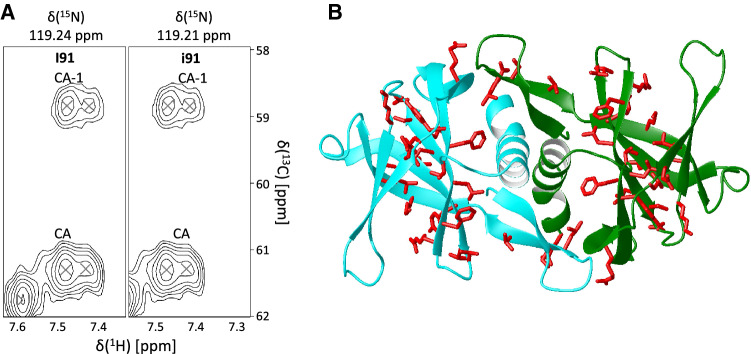
Peak duplications in the spectrum of nsp9. **a** A representative duplicated strip (Ile91) from the HNCA spectrum. We indicated the main species in capital letters, the alternative one in lowercase. **b** Mapping of the duplicated resonances on the X-ray structure (PDB code 6w9q). None of the affected residues is directly involved in the dimer interface

During the assignment process, it became clear that some residues could not be assigned in sample A. Most of the tracing could be done using the spectrum of sample B but some strips were duplicated (Fig. 3a). We could easily identify 30 residues for which splitting was evident. We reasoned that resonance duplication could either result from the co-presence of monomer and dimer, two different dimeric forms or a dimer and a tetramer (possibly in equilibrium). The monomer to dimer possibility was ruled out because it would not be consistent with all the previous literature. The presence of a tetramer is unlikely because the molecular weight would be at the limit of the NMR detection under these conditions (ca. 45 kDa). Investigation of the X-ray structure (6w9q) showed the duplicated residues were distributed along the whole sequence and none of them was observed for residues directly in the interface (Fig. 3b). It is thus possible that resonance duplication reflected the presence of two dimers with slightly different structures or different interfaces, trapped during purification. Structural differences could for instance result from an asymmetrical assembly of the dimer in one of the two forms (Nooren et al. 1999), different interfaces (Sutton et al. 2004) or domain swapping (Liu and Eisenberg 2002). Domain swapping could also explain the differences observed between samples A and B and would be consistent with a rather different distribution of chemical shifts in the C-terminus of the two samples. Identification of the second species will require, in the future, an NMR high resolution structure determination backed up by independent evidence as it could come, for instance, from H/D exchange detected by mass spectrometry and/or filtered NOEs from labelled/unlabelled mixed samples. More work will be required in the future to clarify this point.

## Note in publication

After our manuscript was submitted, another paper was published describing the NMR spectrum assignment of SARS-CoV2 nsp9 (Buchko GW, Zhou M, Craig JK, Van Voorhis WC, Myler PJ. (2021) Backbone chemical shift assignments for the SARS-CoV-2 non-structural protein Nsp9: intermediate (ms–μs) dynamics in the C-terminal helix at the dimer interface. Biomol NMR Assign. Jan 4:1–10). On the whole, the data are in excellent agreement. The spectrum reported corresponds to that of our sample A.

## Data Availability

The assignments of both sample A and B were deposited in BMRB with Accession Numbers 50622 and 50621 respectively.
